# Activated amelogenin Y-linked (*AMELY*) regulation and angiogenesis in human hepatocellular carcinoma by biocomputation

**DOI:** 10.3892/ol.2013.1122

**Published:** 2013-01-10

**Authors:** LIANXIU QI, LIN WANG, JUXIANG HUANG, MINGHU JIANG, HAIZHEN DIAO, HUILEI ZHOU, XIAOHE LI, ZHENFU JIANG

**Affiliations:** 1 Biomedical Center, School of Electronic Engineering, Beijing University of Posts and Telecommunications, Beijing 100876;; 2 Lab of Computational Linguistics, School of Humanities and Social Sciences, Tsinghua University, Beijing 100084;; 3 Shandong Longkou Yimin Molecular Drug Network Research and Development Center, Shandong 265700, P.R. China

**Keywords:** activated amelogenin Y-linked (*AMELY*) upstream regulation network, Wnt signaling, calcium signaling, cell growth, angiogenesis, theoretical analysis, hepatocellular carcinoma

## Abstract

In the present study, a comparison of the biological processes and gene ontology (GO) in human hepatocellular carcinoma (HCC) with high expression (fold change ≥2) of amelogenin Y-linked *(AMELY)*-activated upstream regulation networks with non-tumor hepatitis/cirrhotic tissues (HBV or HCV infection) with low expression of activated networks was performed. The principle biological processes involved in non-tumor hepatitis/cirrhotic tissues include positive regulation of mismatch repair, regulation of transcription from RNA polymerase II promoters, negative regulation of cell-cell adhesion, protein ubiquitinatin and protein catabolism. The main biological processes involved in the development of HCC include positive regulation of calcium ion transport into the cytosol, cell proliferation, DNA replication, fibroblast proliferation, immune response, microtubule polymerization and protein secretion. Specific transcription from RNA polymerase II promoters, regulation of angiogenesis, cell growth, protein metabolism, Wnt receptor signaling pathways, negative regulation of endothelial cell differentiation, microtubule depolymerization, peptidase activity and progression through the cell cycle are also involved. Positive regulation of transcription is involved in both processes. An activated *AMELY*-coupled upstream positive regulation of immune response-mediated protein secretion to Wnt signaling and calcium into cytosol-induced regulation of cell growth and angiogenesis in HCC is proposed. The *AMELY* upstream regulation molecular network model was constructed with *BUB1B, CST6, ESM1, HOXA5, LEF1, MAPT, MYBL2, NOTCH3, PLA2G1B, PROK1, ROBO1, SCML2* and *UBE2C* in HCC from a Gene Expression Omnibus (GEO) dataset by gene regulation network inference methods and our programming methods.

## Introduction

Amelogenin Y-linked *(AMELY)* has been identified to be one of the significantly highly expressed (fold change ≥2) genes in human hepatocellular carcinoma (HCC). Systems-computational analysis was used to elucidate the function and mechanism of *AMELY*-activated upstream regulation networks in HCC compared with low or non-tumor hepatitis/cirrhotic tissues (HBV or HCV infection) from GEO dataset GSE10140-10141 (1).

The possible correlations among immune response, protein secretion, Wnt signaling and calcium release into the cytosol with cancer or tumor formation have been investigated in several studies. Examples include the tetraspanins and the immune response against cancer (2); induction of tumor-specific immune response by gene transfer of Hsp70 cell-penetrating peptide fusion protein and tumors in mice (3); *in situ* immune responses after neoadjuvant chemotherapy for breast cancer predicting survival (4); human tumor cells killed by anthracyclines inducing a tumor-specific immune response (5); keratin 17 promoting epithelial proliferation and tumor growth by polarizing the immune response in skin (6); interleukin-10, but not interleukin-18, possibly being associated with the immune response against well-differentiated thyroid cancer (7); CXCR7 protein expression correlating with elevated mmp-3 secretion in breast cancer cells (8); heat shock cognate 70 protein secretion as a new growth arrest signal for cancer cells (9); overexpression of p53 protein and local hGH, IGF-I, IGFBP-3, IGFBP-2 and PRL secretion by human breast cancer explants (10); restoration of full-length APC protein in SW480 colon cancer cells inducing exosome-mediated secretion of DKK-4 (11); cancer cell secretion of the DAMP protein HMGB1 supporting progression in malignant mesothelioma (12); targeting Wnt signaling in colon cancer stem cells (13); protein cross-talk in CD133^+^ colon cancer cells indicating activation of the Wnt pathway and upregulation of SRp20 that is potentially involved in tumorigenicity (14); miRNA-34 intrinsically linking p53 tumor suppressor and Wnt signaling (15); the Wnt/β-catenin pathway regulating self-renewal of cancer stem-like cells in human gastric cancer (16); a serrated colorectal cancer pathway predominating over the classic Wnt pathway in patients with hyperplastic polyposis syndrome (17); insights from studies with oral cleft genes suggesting associations between Wnt pathway genes and risk of oral cancer (18); and hydrogen sulfide increasing calcium-activated potassium (BK) channel activity of rat pituitary tumor cells (19). The function and mechanism of the high expression of the *AMELY*-activated upstream regulation network in HCC is not known and remains to be elucidated.

The aim of this study was to compare the biological processes and occurrence numbers of gene ontology (GO) in non-tumor hepatitis/cirrhotic tissues (HBV or HCV infection) with low expression of *AMELY* upstream regulation networks and the corresponding HCC tissue with high expression (fold change ≥2) of *AMELY. AMELY*-activated upstream regulation molecular networks in non-tumor hepatitis/cirrhotic tissues and HCC were constructed. A further aim was to identify the *AMELY* upstream regulation molecular network involved in HCC.

## Materials and methods

### Materials

Microarrays from of 6,144 genes from 25 non-tumor hepatitis/cirrhotic tissues and 25 HCC patients were used for analyzing the possible AMELY-activated upstream regulation networks of HCC based on GEO dataset GSE10140-10141 (http://www.ncbi.nlm.nih.gov/geo/query/acc.cgi?acc=GSE10140 and http://www.ncbi.nlm.nih.gov/geo/query/acc.cgi?acc=GSE10141). The raw microarray data were pre-processed using log base 2.

### Methods

A total of 225 molecules with a significantly high expression (fold change ≥2) in HCC were identified for studying the function and mechanism of *AMELY*-activated upstream regulation networks in **HCC by systems-computational analysis** of difference with low or non-tumor hepatitis/cirrhotic tissues using significant analysis of microarrays (SAM; http://www-stat.stanford.edu/~tibs/SAM/) (20). We selected the two-class paired data and a minimum fold change ≥2 under the false-discovery rate of 0%.

The *AMELY*-activated upstream regulation network of HCC was analyzed using the Molecule Annotation System, MAS (CapitalBio Corporation, Beijing, China). The primary databases of MAS integrated various well-known biological resources, such as Gene Ontology (http://www.geneontology.org), KEGG (http://www.genome.jp/kegg/), BioCarta (http://www.biocarta.com/), GenMapp (http://www.genmapp.org/), HPRD (http://www.hprd.org/), MINT (http://mint.bio.uniroma2.it/mint/Welcome.do), BIND (http://www.blueprint.org/), Intact (http://www.ebi.ac.uk/intact/), UniGene (www.ncbi.nlm.nih.gov/UniGene) and OMIM (http://www.ncbi.nlm.nih.gov/entrez/query.fcgi?db=OMIM).

Biological processes and occurrence numbers of GO in non-tumor hepatitis/cirrhotic tissues (HBV or HCV infection) with activated low expression of AMELY upstream regulation networks and the corresponding HCC tissues with high expression (fold change ≥2) were identified and computed.

*AMELY*-activated upstream regulation molecular networks in non-tumor hepatitis/cirrhotic tissues and HCC were constructed by GRNInfer (21) and published studies (22–36), and illustrated by GVedit tool, respectively.

## Results

**Figure 1:**
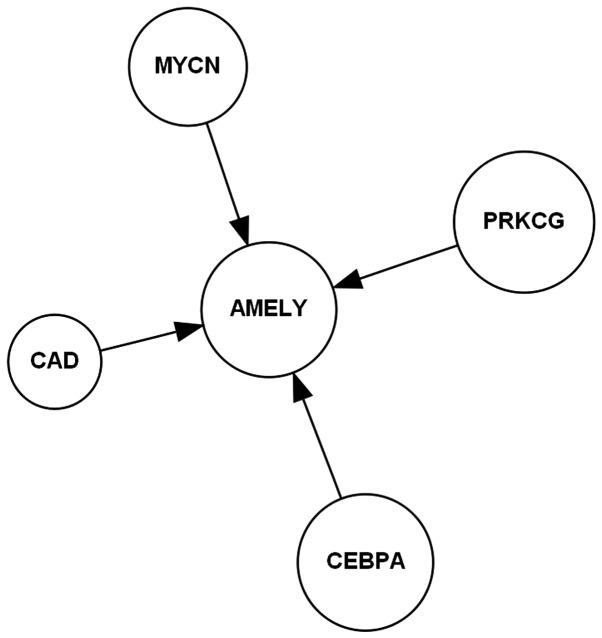
Amelogenin Y-linked (*AMELY)*-activated upstream regulation molecular network in non tumor hepatitis/cirrhotic tissues by GRNInfer and our programming.

**Figure 2:**
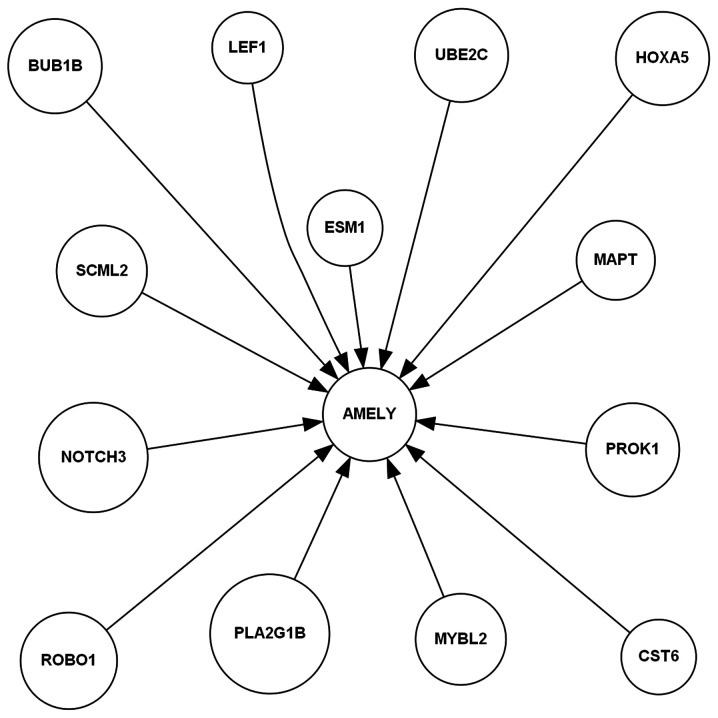
Amelogenin Y-linked (*AMELY)*-activated upstream regulation molecular network in human hepatocellular carcinoma (HCC) by GRNInfer and our programming.

The biological processes of AMELY-activated upstream regulation networks in non-tumor hepatitis/cirrhotic tissues and HCC are presented in Tables I and II, respectively. The *AMELY*-activated upstream regulation molecular network consisted of *CAD, CEBPA, MYCN* and *PRKCG* in non-tumor hepatitis/cirrhotic tissues, as shown in Fig. 1. The *AMELY*-activated upstream regulation molecular network included *BUB1B, CST6, ESM1, HOXA5, LEF1, MAPT, MYBL2, NOTCH3, PLA2G1B, PROK1, ROBO1, SCML2* and *UBE2C* in HCC, as shown in Fig. 2.

## Discussion

The aim of this study was to elucidate the function and mechanism of *AMELY*-activated upstream regulation networks in HCC using systems-computational analysis of differences and similarities with non-tumor hepatitis/cirrhotic tissues (HBV or HCV infection). Biological processes and occurrence numbers of GO in non-tumor tissues with activated low expression of *AMELY* upstream regulation networks and the corresponding HCC tissues with high expression (fold change ≥2) were identified and computed (Tables I and II).

We analyzed and compared the biological processes and occurrence numbers of GO in HCC tissues with high expression (fold change ≥2) of the *AMELY*-activated upstream regulation network and the corresponding non-tumor hepatitis/cirrhotic tissues with low expression of the activated network. The biological processes identified solely in non-tumor hepatitis/cirrhotic tissues included negative regulation of cell-cell adhesion, transcription from RNA polymerase II promoter, protein ubiquitination and protein catabolism, as well as positive regulation of mismatch repair. The processes found only in HCC consisted of negative regulation of endothelial cell differentiation, microtubule depolymerization, peptidase activity, progression through the cell cycle, positive regulation of calcium ion transport into the cytosol, cell proliferation, DNA replication, fibroblast proliferation, immune response, microtubule polymerization, protein secretion, and specific transcription from RNA polymerase II promoter. There was also regulation of angiogenesis, cell growth, protein metabolism and the Wnt receptor signaling pathway. The processes common to both included positive regulation of transcription. It was therefore postulated that *AMELY*-activated coupling upstream positively regulates the immune response-mediated protein secretion to Wnt signaling and calcium into cytosol-induced regulation of cell growth and angiogenesis in HCC.

The correlations between regulation of immune response, protein secretion, Wnt signaling and calcium movement into the cytosol with regulation of cell growth or angiogenesis have been reported. Examples include calcium store sensor stromal-interaction molecule 1-dependent signaling playing an important role in cervical cancer growth, migration and angiogenesis (37); blockade of Wnt signaling inhibiting angiogenesis and tumor growth in HCC (38); enhancement of the recognized Wnt/β-catenin signaling activity by HCV core protein promoting cell growth of HCC cells (39); porous membrane substrates offering better niches to enhance the Wnt signaling and promote human embryonic stem cell growth and differentiation (40); effect of dietary tea polyphenols on growth performance and cell-mediated immune response of post-weaning piglets under oxidative stress (41); Wnt inhibitory factor 1 inducing apoptosis and inhibiting cervical cancer growth, invasion and angiogenesis *in vivo*(42); Rspo1/Wnt signaling promoting angiogenesis via Vegfc/Vegfr3 (43); bone morphogenetic protein 2 inducing pulmonary angiogenesis via Wnt-β-catenin and Wnt-RhoA-Rac1 pathways (44); and the correlation between angiogenesis and the immune response in carcinogenesis and the progression of malignant disease (45).

**Figure 3:**
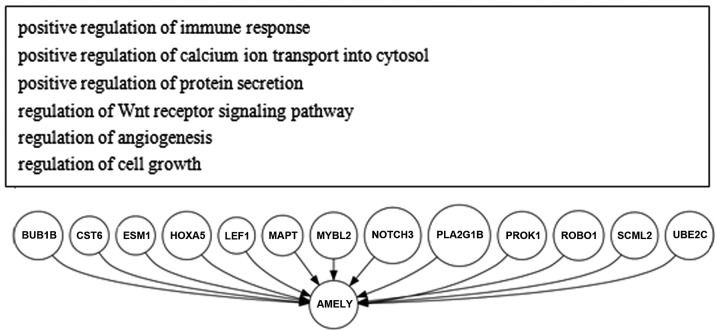
A model of the activated amelogenin Y-linked (*AMELY*) upstream regulation molecular network in human hepatocellular carcinoma (HCC) from different comparisons with the corresponding activated gene ontology (GO) molecular network of non-tumor hepatitis/cirrhotic tissues.

*AMELY*-activated upstream regulation molecular networks in non-tumor hepatitis/cirrhotic tissues and HCC were constructed (Figs. 1 and 2). We further constructed a model of the *AMELY* upstream regulation molecular network in HCC only, which included *BUB1B*, *CST6*, *ESM1*, *HOXA5*, *LEF1*, *MAPT*, *MYBL2*, *NOTCH3*, *PLA2G1B*, *PROK1*, *ROBO1*, *SCML2* and *UBE2C*, by comparison with the corresponding activated GO molecular network of non-tumor hepatitis/cirrhotic tissues (Fig. 3).

In summary, the biological processes and occurrence numbers of GO in HCC tissue with high expression (fold change ≥2) of *AMELY*-activated upstream regulation network were compared with those of the corresponding non-tumor hepatitis/cirrhotic tissues with low expression of the activated network. The biological processes in the non-tumor hepatitis/cirrhotic tissues included positive regulation of mismatch repair, regulation of transcription from RNA polymerase II promoter, negative regulation of cell-cell adhesion, protein ubiquitination and protein catabolism. The processes in HCC consisted of positive regulation of calcium ion transport into the cytosol, cell proliferation, DNA replication, fibroblast proliferation, immune response, microtubule polymerization, protein secretion and specific transcription from RNA polymerase II promoter; regulation of angiogenesis, cell growth, protein metabolism and the Wnt receptor signaling pathway; and negative regulation of endothelial cell differentiation, microtubule depolymerization, peptidase activity and progression through the cell cycle. A common factor included positive regulation of transcription. Activated *AMELY* coupling upstream positive regulation of immune response-mediated protein secretion to Wnt signaling and calcium into cytosol-induced regulation of cell growth and angiogenesis network in HCC was proposed. The *AMELY* upstream regulation molecular network model was constructed, including *BUB1B, CST6, ESM1, HOXA5, LEF1, MAPT, MYBL2, NOTCH3, PLA2G1B, PROK1, ROBO1, SCML2* and *UBE2C*, in HCC from a GEO dataset by gene regulation network inference methods and computational programming.

## Figures and Tables

**Table I:** Biological processes of *AMELY* upstream regulation network in non-tumor hepatitis/cirrhotic tissues using our programming.

Biological process	Occurrence of GO term
Negative regulation of cell-cell adhesion	1
Negative regulation of protein catabolism	1
Negative regulation of protein ubiquitination	1
Positive regulation of mismatch repair	1
Positive regulation of transcription	1
Regulation of transcription from RNA polymerase II promoter	1

GO, gene ontology; *AMELY*, amelogenin Y-linked.

**Table II:** Biological processes of *AMELY* upstream regulation network in human HCC using our programming.

Biological process	Occurrence of GO term
Negative regulation of endothelial cell differentiation	1
Negative regulation of microtubule depolymerization	1
Negative regulation of peptidase activity	1
Negative regulation of progression through cell cycle	1
Positive regulation of calcium ion transport into cytosol	1
Positive regulation of cell proliferation	1
Positive regulation of DNA replication	1
Positive regulation of fibroblast proliferation	1
Positive regulation of immune response	1
Positive regulation of microtubule polymerization	1
Positive regulation of protein secretion	1
Positive regulation of specific transcription from RNA polymerase II promoter	1
Positive regulation of transcription	1
Regulation of angiogenesis	1
Regulation of cell growth	1
Regulation of protein metabolism	1
Regulation of Wnt receptor signaling pathway	1

GO, gene ontology; *AMELY*, amelogenin Y-linked; HCC, hepatocellular carcinoma.
